# Quality Control Strategies for Differentiation of *Kalanchoe* Species

**DOI:** 10.1002/pca.3525

**Published:** 2025-03-11

**Authors:** Evelyn A. de Andrade, Isadora Machinski, Valter P. de Almeida, Sarah A. Barr, Wilmer H. Perera, Jane Manfron, Flávio L. Beltrame, R. Thomas Williamson, Wendy K. Strangman

**Affiliations:** ^1^ State University of Ponta Grossa Ponta Grossa Paraná Brazil; ^2^ University of North Carolina Wilmington Wilmington North Carolina USA; ^3^ CAMAG Scientific, Inc Wilmington North Carolina USA

**Keywords:** HPTLC, natural products, pharmacobotanical, quality control, UPLC‐MS

## Abstract

*Kalanchoe* species products are commercially available in local markets and by internationally accessible online retailers and may exhibit quality issues because of misidentification from similar common names and anatomical similarities among related species used as feedstock. This study proposes an approach using UPLC‐MS/MS^E^ and HPTLC, coupled with morphoanatomical analysis to establish chemical composition pattern data for five *Kalanchoe* species. Subsequently, the methods were validated by analyzing commercial products purported to contain 100% *Kalanchoe* extract. UPLC‐MS/MS^E^ and HPTLC profiles demonstrated that quercetin and kaempferol derivatives were identified as the primary flavonoids in genuine plant extracts. Chemometric analysis showed clear differences in chemical profiles and no similarities between the *Kalanchoe* plant extracts and commercial products. Different patterns of anticlinal epidermal cell walls and midrib of the leaves and shape and arrangement of the vascular bundles in the petiole were the primary micro‐morphological differences observed. Evaluation of commercial samples revealed that products labeled as containing *Kalanchoe* did not match the pharmacobotanical analysis nor the chemical composition of the species. These methods can be considered important tools for quality control in commercial products derived from *Kalanchoe* species.

## Abstract 2

This study addresses quality control challenges in *Kalanchoe* species due to common names and anatomical similarities. Using a multidisciplinary approach involving UPLC‐MS/MS^E^, HPTLC, and morphoanatomical analysis, this research differentiates five *Kalanchoe* species, identifying quercetin and kaempferol derivatives as key component flavonoids. Chemometric and anatomical analyses revealed significant differences between authentic plant extracts and commercial products labeled as *Kalanchoe*. These methods are effective tools for ensuring species identification and quality control in herbal products.

## 1 Introduction

Herbal products are found under a variety of designations in accordance with the regulatory frameworks of their respective countries. In Brazil, these products are categorized as either herbal medicines or traditional herbal products [1], whereas in the United States of America, they are commonly sold as dietary supplements under the Dietary Supplement Health and Education Act of 1994 [2]. The variance in international regulatory standards often results in disparities in enforcement, which can affect the quality of medicinal plants and their commercial products, impacting the authenticity of herbal raw materials in the global market. Adulteration, a prevalent issue within this sector, is frequently exacerbated by the morphological similarities among related species and the use of their common names. These practices in the herbal medicine industry may compromise the therapeutic effectiveness of these products and, in certain instances, pose risks of intoxication to consumers [3].

An example of a widely consumed botanical, particularly in traditional medicine in regions of Africa, Asia, and South America, is the *Kalanchoe* genus [4]. The difficulty in distinguishing species within this genus has been previously highlighted because of inconsistencies in vernacular names and morphological similarities, which often lead to misidentification of their taxonomic characteristics. For instance, *Kalanchoe daigremontiana* Raym.‐Hamet & H.Perrier and *Kalanchoe* x *houghtonii* D.B.Ward, which exhibit notable morphological similarities, are frequently referred to by the same vernacular name, “mother‐of‐thousands” or “mother‐of‐millions.” This confusion complicates their use in folk medicine and the medicinal/supplementary market. Similarly, *Kalanchoe crenata* (Andrews) Haw., *Kalanchoe marmorata* Baker, and *Kalanchoe pinnata* (Lam.) Pers., which are sometimes collectively called “Bryophyllum” or “life‐plant,” share overlapping uses in various health applications, reflecting their collective popularity in treating diverse ailments. These instances highlight the need for accurate identification and understanding of each species, to assess their safety and efficacy, and to fully harness their therapeutic potential. Botanical analysis for evaluating these herbal materials is a crucial step in ensuring differentiation, quality, safety, and consistency.

Chemical identification of botanicals can also be used as an important tool to complement the taxonomic identification of these herbal materials [5]. High‐performance thin‐layer chromatography (HPTLC) is a compendial and gold standard technique in the United States for botanical ID testing. It serves as a powerful tool for rapidly comparing multiple samples in parallel, assessing biochemical activity, detecting adulteration, monitoring purity and stability, and identifying and quantifying marker compounds in both raw materials and finished products [6, 7, 8, 9, 10]. Another technique for analyzing botanicals is ultra‐performance liquid chromatography coupled with high‐resolution mass spectrometry (UPLC‐MS), which is often used in connection with untargeted metabolomic profiling for characterizing the chemical composition of plant matrices and aiding in the identification of bioactive molecules and markers [11, 12, 13].

Combining microscopic examination with chemical analysis can provide comprehensive insights into the species' chemometrics [14]. In this investigation, a multidisciplinary approach including microanatomical analysis, untargeted UPLC‐MS/MS^E^ metabolite profiling, and HPTLC fingerprinting was employed to characterize and differentiate five *Kalanchoe* species. These studies can serve as a baseline for developing quality control tools for commercial product evaluation.

## 2 Material and Methods

### 2.1 Collection and Identification of Plant Materials

Fresh aerial vegetative parts from five species of the genus *Kalanchoe* were collected in Ponta Grossa, Brazil (25°5′38″ S 50°12′34″ W). The flowering plants were used for the preparation of herbarium specimens and were identified by Dr. Gustavo Heiden, from The Brazilian Agricultural Research Corporation (EMBRAPA), and Dr. Vijayasankar Raman, from the National Center for Natural Products Research, The University of Mississippi (NCNPR), as *K. crenata* (Andrews) Haw., *K. daigremontiana* Raym.‐Hamet & H.Perrier, *K. marmorata* Baker, *K. pinnata* (Lam.) Pers., and *K*. x *houghtonii* D.B.Ward. The specimens were registered and deposited by Dr. Rosângela Capuano Tardivo, from the State University of Ponta Grossa Herbarium, under registration numbers 22929, 22778, 22779, 23063, and 22808, respectively. Access to botanical materials was registered in the Brazilian National System for the Management of Genetic Heritage and Associated Traditional Knowledge (Sistema Nacional de Gestão do Patrimônio Genético e do Conhecimento Tradicional Associado—SISGEN), under registration AFDD6B4.

### 2.2 Preparation of the Plant Materials and Extracts

Fresh leaves and stems of each species were collected, crushed, and extracted with distilled water using turbo extraction (1:10, w/v, 5 min). Afterward, the extracts were filtered and freeze‐dried. Capsules from commercially available products were purchased from three different companies claiming to contain 100% *Kalanchoe* extract (one sample labeled as *K. daigremontiana* and two samples labeled as *K. pinnata* ). These samples were processed using an identical procedure to that used for the fresh leaf and stem extracts. The extracts and commercial products were diluted to a concentration of 0.1 mg/mL (MeCN: H_2_O, 1:1, v/v) prior to UPLC‐MS/MS^E^ analysis and 20 mg/mL (H_2_O) for HPTLC analysis.

### 2.3 Botanical Evaluation and Powder Analysis

The samples (leaves and stems) were fixed for five days in a solution of formalin, acetic acid, and alcohol 70% (FAA 5:5:90, v/v/v) [15]. Afterward, they were washed with purified water and stored in ethanol 70% (v/v) [16]. For the microscopic examination of plant material, paradermal and transverse sections were prepared. These materials were stained with astra blue and basic fuchsin [17], as well as toluidine blue [18] to obtain semipermanent slides, which were mounted on glass slides with glycerol 50% (v/v). The slides were immediately photographed and analyzed under a light microscope coupled with a photographic camera (Olympus CX31 model with C 7070 control unit) for a detailed description of leaf and stem anatomy. To analyze the powder, a meticulous microscopic analysis was employed by preparing slides of the powder material of the capsules from three commercially available products purchased from two different companies claiming to contain 100% of *Kalanchoe* species: (1) *K. daigremontiana* commercial product (KDCP); (2) *K. pinnata* commercial product A (KPCPA); and *K. pinnata* commercial product B (KPCPB). In this analysis, a combination of astra blue and basic fuchsin, and safranin [19] were used as structural reagents, as well as iodine solution to detect starch grains [16]. This approach combined botanical knowledge with pharmacognosy techniques to ensure the authenticity and purity of the herbal products.

### 2.4 HPTLC Analysis

#### 2.4.1 Standards Preparation

Stock solutions of quercetin, rutin, chlorogenic acid, and kaempferol were prepared at 1 mg/mL each in methanol. Chlorogenic acid, quercetin, and kaempferol were further diluted to 200 μg/mL, whereas rutin was prepared at 400 μg/mL. The universal HPTLC mix (UHM) was used as a system suitability test (SST) and prepared according to the literature [20].

#### 2.4.2 Application and Development

HPTLC analyses were conducted using a CAMAG HPTLC PRO system (Wilmington, NC) equipped with four modules. Plates were set on carriers and stored in a Plate Storage PRO module and transported into the Application PRO module for further application. Sample (10 μL), SST, and standard (2 μL) solutions were applied onto a 20 × 10 cm HPTLC silica gel 60 F254 plate (Merck, Germany) according to the United States Pharmacopeia (USP) general chapter <203> [21]. After application, the plate was transported automatically through conveyors into the Development PRO module and developed with a freshly prepared solution of *n*‐butyl acetate, methanol, water, and formic acid (7.5:2:1:1, v/v/v/v). The plate was activated for 10 min by blowing air from a bottle containing a saturated solution of MgCl_2_ into the chamber. The gas phase was generated in the conditioning tubing with the mobile phase at 25% pump power and sent to the chamber in a controlled way for plate conditioning from 30 to 70 mm developing distance. Next, the plate was dried inside the chamber and visualized under short wavelength UV (254 nm) using the TLC Visualizer 2 module.

#### 2.4.3 Post‐Chromatographic Derivatization and Visualization

The plate was heated at 100°C for 90 s and then derivatized with 1.5 mL of natural product (NP) reagent (1 g of 2‐aminoethyl diphenylborinate dissolved in 100 mL of methanol) under reduced pressure using a PRO Derivatization module with Nozzle 1 and spraying Level 3. Images were recorded using a TLC Visualizer 2 under longwave (UV 366 nm) in reflection and transmission mode. Another detection mode was added by spraying 1.8 mL of anisaldehyde reagent (85 mL of ice‐cooled methanol was slowly mixed with 10 mL of acetic acid and 5 mL of sulfuric acid). Allowing the mixture to cool down to room temperature, 0.5 mL of anisaldehyde (ρ‐methoxy benzaldehyde) was added under reduced pressure using the PRO Derivatization module with Nozzle 2 and spraying Level 2; then, the plate was heated at 100°C for 90 s. Images were recorded under 366 nm UV.

### 2.5 UPLC‐MS/MS^E^ Analysis

UPLC‐MS/MS^E^ data for aqueous extracts of *Kalanchoe* species were acquired using a Waters I‐Class UPLC coupled with a Waters G2‐XS Quadrupole Time of Flight (Q‐TOF) Mass Spectrometer. Data were collected in both ESI positive and negative modes. Chromatographic separation was carried out on an Acquity UPLC BEH C18 1.7 μm, 2.1 × 100 mm reversed‐phase column with 0.1% formic acid in water (v/v) (A) and 0.1% formic acid in acetonitrile (v/v) (B) as a binary mobile phase. All samples were analyzed using a gradient elution program, starting at 15% B, and increasing to 30% B over 4 min, maintained for 3 min further increasing to 100% B until 8.2 min, and then re‐equilibrating to initial conditions by 10 min. The flow rate was set at 0.45 mL/min. The sample injection volume was 3 μL, and the column temperature was maintained at 30°C. The mass spectrometer was equipped with an ESI source and operated in both positive and negative ion modes. The scanning mass range was *m/z* 50–1800. The MS^E^ collision energy range was ramped between 35 and 45 V and the cone voltage was maintained at 40 V. Triplicate samples were prepared from each plant extract and vials were randomized prior to sampling. Lock mass correction information was acquired for each sample with a continuous infusion of a 0.2 ng/mL solution of leucine enkephalin at a flow rate of 10 μL/min, generating a reference ion for positive ion mode ([M + H]^+^ = 556.2771) and negative ion mode ([M − H]^−^ = 554.2615) to ensure accuracy during the MS analysis. A mass correction was applied post‐acquisition in the Waters Progenesis QI software (version 3.0.3).

### 2.6 Data Processing and Analysis

UPLC‐MS/MS^E^ data were generated using MassLynx (version 4.2). Raw data were then imported into the software package Progenesis QI. Here, spectra were aligned, and peaks were deconvoluted. The resulting mass feature set was further filtered through the removal of features with a maximum abundance in the experimental control samples. Features were also excluded if they had chromatographic retention time less than 0.75 min or greater than 6 min, and if they had a maximum ion abundance less than 1000. Finally, features were removed if their ANOVA *p*‐value was greater than 0.05. All data were normalized to the summed total ion intensity per chromatogram, and principal component analysis (PCA) was performed.

## 3 Results and Discussion

### 3.1 Pharmacobotanical Analysis

**FIGURE 1:**
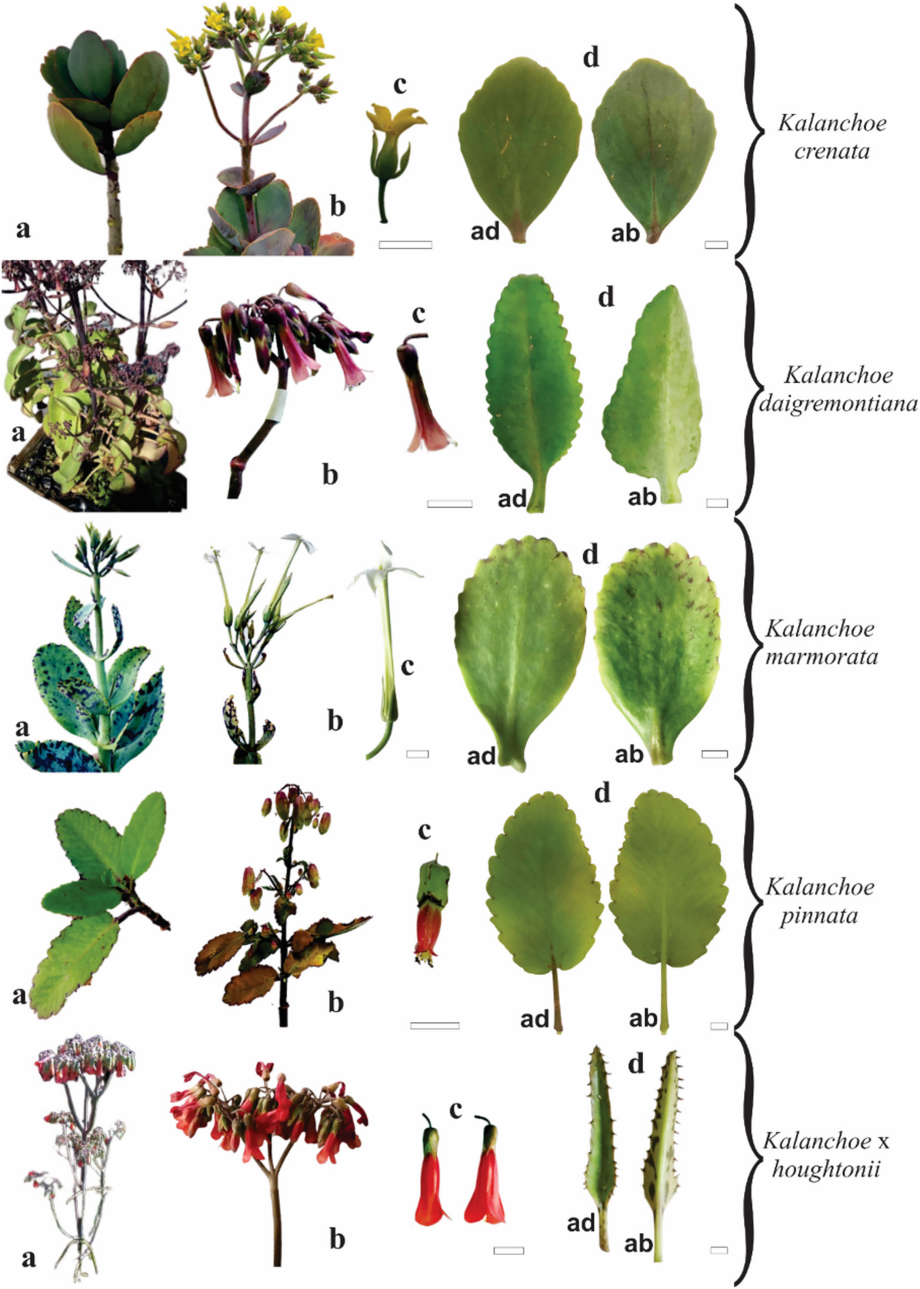
Morphological aspects of five *Kalanchoe* species. Aerial parts (a); terminal inflorescence (b); flowers (c); leaves (d). ad: adaxial side; ab: abaxial side. Scale bar: 1 cm.

All five *Kalanchoe* species analyzed in this study are succulent, glabrous, smooth, single‐veined, and exhibit opposite‐crossed phyllotaxis (Figure 1a). The flowering of all these species can be observed during the winter season when the main peduncle elongates vertically (Figure 1b,c). Although all species possess leaves with a general bright green coloration, key differences lie in their size, shape, apex, basis, and margin characteristics (Figure 1d and Table 1). Particularly notable are the distinctive spots on *K*. x *houghtonii* and *K. marmorata* , and the varying leaf shapes and sizes across these species. In addition to the leaf morphology, in the flowers, important characteristics for their differentiation are highlighted, such as the type of inflorescence (terminal or corymbiform), color (ranging from white to red), size (varying from 2 to 10 cm in length), and shape (star or bell‐like). These features highlight the main differences among these species and aid greatly in their identification and classification. Figure 1 and Table 1 provide detailed descriptions of the morphological aspects of flowers and leaves of the five *Kalanchoe* species.

**TABLE 1:** Morphological aspects for differentiation of five *Kalanchoe* species.

Species	Leaves							Flowers			
	Color	Size (cm)		Shape	Apex	Base	Margin	Inflorescence Aspect	Color	Size (cm)	Shape
		Length	Width							Length	
KC	Bright green	5–10	3–6	Obovate to oval	Obtuse	Cuneate	Crenate	Terminal	Yellow	3–5	Star
KD	Bright green	7–15	4–4.5	Oblong to oval	Obtuse	Asymmetric	Crenate	Corymbiform terminal	Pink to purple	2–3	Bell
KM	Bright green, with purplish spots on both sides, predominantly on the abaxial	5–10	3–6	Obovate to oval	Rounded	Attenuated	Crenate	Terminal	White	6–10	Star
KP	Bright green	5–10	3–6	Obovate to oval	Obtuse	Truncated	Crenate	Corymbiform terminal	Pink	2–3	Bell
KH	Dark green, with brown‐purple spots on the abaxial surface	7–10	3–4	Lanceolate	Acuminate	Attenuated	Dentate	Corymbiform terminal	Orange to red	3–5	Bell

Abbreviations: KC: *Kalanchoe crenata* ; KD: *Kalanchoe daigremontiana* ; KM: *Kalanchoe marmorata* ; KP: *Kalanchoe pinnata* ; KH: *Kalanchoe* x *houghtonii*.

**FIGURE 2:**
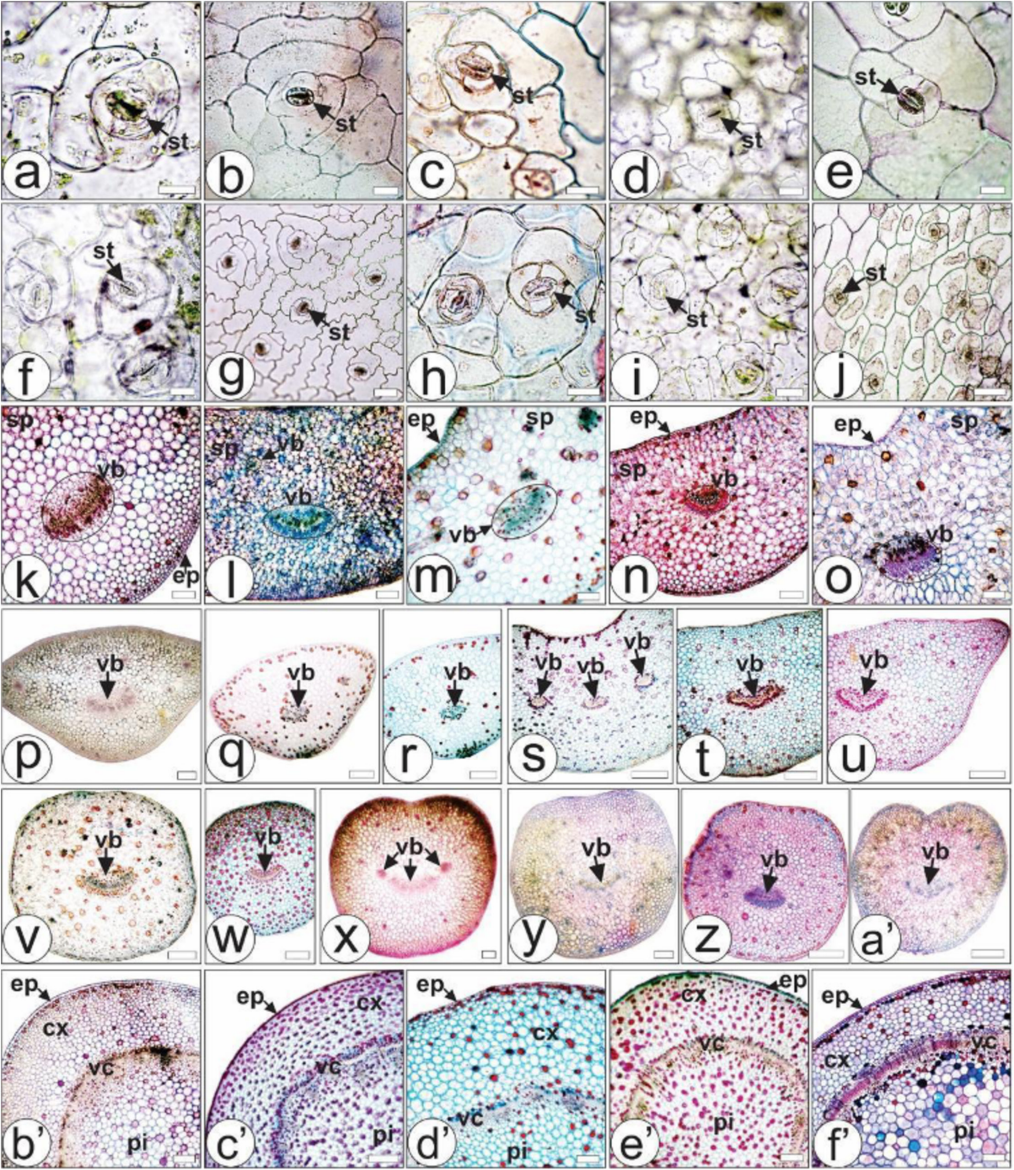
Anatomical aspects of five *Kalanchoe* species by light microscopy. *K. crenata* (a,f,k,p–r,b′), *K. daigremontiana* (b,g,l,s–u,c′), *K. marmorata* (c,h,m,d′), *K. pinnata* (d,i,n,v–x,e′), *K.* x *houghtonii* (e,j,o,y,z,a′,f′). Adaxial surface of leaf (a–e); abaxial surface of leaf (f–j); leaf blade in cross‐section (a–o); petiole distal region in cross‐section (p,s,v,y), petiole medial region in cross‐section (q,t,v,z), and petiole proximal region in cross‐section (r,u,x,a′); stem in cross‐section (b′–f′). Astra blue and basic fuchsine (a–k,m–x,y,z,a′–f′); blue toluidine (l,y,a′). cx: cortex; ep: epidermis; pi: pith; sp: spongy parenchyma; st: stomata; vb: vascular bundle; vc: vascular cylinder. Scale bar: 25 μm (a,c,d,f,h–j,); 50 μm (b,e,g); 125 μm (y); 250 μm (k–o,z,a′–f′); 500 μm (p–x).

Detailed anatomical aspects of the five *Kalanchoe* species are presented in Figure 2. In addition, Table 2 provides detailed descriptions of the anatomical characteristics of leaves of the five *Kalanchoe* species studied. Each species exhibits unique anatomical characteristics in their leaves and petioles, aiding in their identification. The differences lie primarily in the anticlinal epidermal cell walls' patterns and midrib structure for the leaves and in the shape and vascular bundle arrangement for the petioles.

**TABLE 2:** Anatomical aspects for differentiation of five *Kalanchoe* species.

Species	Leaves			Petioles					
	Anticlinal epidermal cell walls		Midrib	Distal		Medial		Proximal	
	Abaxial	Adaxial	Shape	Shape	Vascular bundles	Shape	Vascular bundles	Shape	Vascular bundles
KC	Wavy	Wavy	Bi‐convex	Bi‐convex with 2 lateral wings	1 collateral in open arc	Bi‐convex with 2 lateral wings	1 collateral in open arc	Bi‐convex with 2 lateral wings	1 collateral in open arc
KD	Sinuous	Wavy	Concave‐convex	Concave‐convex, with 2 lateral wings	3 collaterals in open arc	Flat‐convex, with 2 lateral wings	1 collateral in open arc	Flat‐convex, with 2 lateral wings	1 collateral in open arc
KM	Wavy	Wavy	Concave‐convex	Absent	Absent	Absent	Absent	Absent	Absent
KP	Sinuous	Wavy	Flat‐convex	Circular	1 collateral in open arc	Circular	1 collateral in open arc	Concave–convex	1 collateral in open arc and 2 dorsal traces
KH	Straight	Wavy	Concave‐convex	Slight concave–convex	1 collateral in open arc	Circular	1 collateral in open arc	Concave–convex	1 collateral in open arc

Abbreviations: KC: *Kalanchoe crenata* ; KD: *Kalanchoe daigremontiana* ; KM: *Kalanchoe marmorata* ; KP: *Kalanchoe pinnata* ; KH: *Kalanchoe* x *houghtonii*.

These detailed morphological features are crucial for accurate species identification and classification of the *Kalanchoe* species. In the present study, leaf propagules were also observed emerging from the dry structures of the terminal inflorescences after the flowering period. This is one of the propagation methods of *K. daigremontiana* and *K*. x *houghtonii*. This type of flower propagation has not been described in the literature.

The characteristic of *K. daigremontiana* being monocarpic and multiannual, along with terminal inflorescences of small bell‐shaped pink flowers, was previously described [22]. The characteristics of lanceolate and serrated *K*. x *houghtonii* leaves, along with corymbiform inflorescences of pendulous flowers, tetramerous or pentamerous, with dark red coloration, were also observed for this species [23]. Other authors [24, 25, 26] have referred to both species as *K. daigremontiana* , but they provided descriptions and images of *K*. x *houghtonii*. The leaf color characteristics have already been described [22, 27, 28, 29].

Morphological characterization alone may not always be sufficient to determine the authenticity of a plant species, particularly in cases where species within complex genera or similar morphological traits need to be distinguished. Therefore, microscopic analysis plays a crucial role in the authentication process, as anatomical features, especially botanical markers, are key factors in accurately identifying plant species [3]. The anticlinal epidermal cell walls are thin in all species (Figure 2). In the literature, it was found that for *K. pinnata* , the anticlinal epidermal cell walls are sinuous on both surfaces; for *K. crenata* , the anticlinal epidermal cell walls are straight to slightly sinuous on the adaxial surface while appearing sinuous on the abaxial surface; for *Kalanchoe pumila* Baker, the anticlinal cell walls range from straight to wavy, with the abaxial epidermis composed of smaller cells compared with those on the adaxial surface [30, 31]. The shape of the anticlinal epidermal cell walls can be considered a good parameter for differentiation among species in the genus *Kalanchoe*.

The epidermis of leaves, petioles, and stems are uniseriate, with smooth and thin cuticles, and the mesophyll is homogeneous, with spongy parenchyma (Figure 2). The presence of a thin cuticle was also reported for the stem of *K. daigremontiana* [32]. According to literature data, the epidermis of *K*. x *houghtonii* is uniseriate, and the cuticle is thick, smooth to slightly wavy, with distinct striations on subsidiary cells and wax deposits [25, 30]. Additionally, three large bundles were observed [25] in the midribs of *K*. x *houghtonii* leaves, different from what is found in the present study. Transverse sections show thin lateral vascular bundles surrounding the large bundles in the leaf blades.

Stomata of the five species are anisocytic, present on both surfaces of the leaves (described as amphistomatic) (Figure 2). Amphistomatic leaves and anisocytic stomata are common in species of the family Crassulaceae and the genus *Kalanchoe* [25, 30, 31, 33, 34].

In the present study, the stems of all species showed a circular shape, with several layers of collenchyma and a continuous vascular cylinder (Figure 2). No trichomes were found in the species studied. Trichomes are rarely found in the family Crassulaceae [34]. Fifteen species of the genus *Kalanchoe* were studied [32], reporting trichomes in only three species, such as *Kalanchoe beharensis* Drake, *Kalanchoe tomentosa* Baker, and *Kalanchoe caniflora* Adans. Other authors [30] did not observe trichomes on the leaf surface of *K. pumila* .

### 3.2 Chemical Analysis

Chemical analysis is critical for the characterization of botanical powders and chemical extracts as most morphological features are no longer present. Flavonoids and phenolic acids, prevalent secondary metabolites in plants, are often utilized for generating chemical fingerprints for identification. HPTLC stands out among techniques for its increasing application in herbal drug chemotyping for species identification, for which various methods have been established [7, 8, 9, 10].

Data concerning the chemical composition of all *Kalanchoe* species in scientific databases from the last 40 years has yielded reports for 124 compounds, mainly phenolic compounds, and mostly from *K. daigremontiana* and *K. pinnata* [4]. Phenolic compounds are considered important bioactive constituents, responsible for the medicinal properties attributed to the *Kalanchoe* genus. Flavonoids, including glycosyl derivatives of quercetin, patuletin, and kaempferol, have been identified and reported in aqueous, hydroalcoholic, and alcoholic leaf extracts of various *Kalanchoe* species. Specific quercetin derivatives such as quercetin 3‐O‐rhamnopyranoside (quercitrin) and quercetin 3‐O‐α‐L‐arabinopyranosyl (1 → 2) α‐L‐rhamnopyranoside (QAR) are highlighted for their bioactive properties, including antimicrobial, antiviral, anti‐inflammatory, antioxidant, and wound healing effects [4, 35, 36]. Therefore, the HPTLC component of the present study focused specifically on fingerprinting phenolic compounds.

**FIGURE 3:**
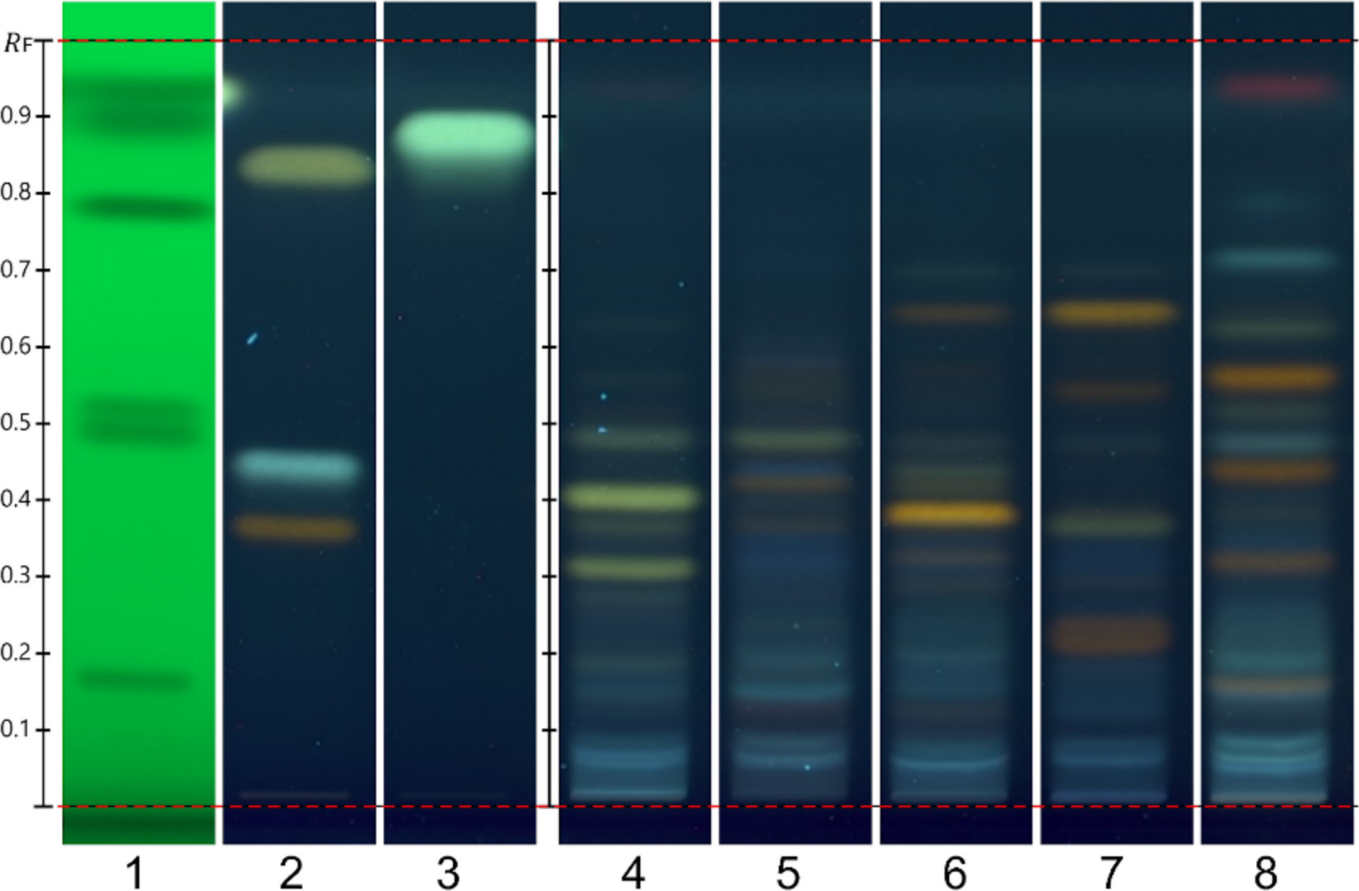
HPTLC profile of *Kalanchoe* species extracts and commercial products under 366 UV light after derivatization with NP reagent. Track 1: SST; Track 2: rutin, chlorogenic acid, and quercetin (with increasing *R*_F_); Track 3: kaempferol; Track 4: aqueous extract of *K. daigremontiana* ; Track 5: aqueous extract of *K.* x *houghtonii*; Track 6: aqueous extract of *K. pinnata* ; Track 7: aqueous extract of *K. marmorata* ; Track 8: aqueous extract of *K. crenata* . Contrast 2.0.

Recently, a method that separates phenolic acids, aglycones, and glycosidic flavonoids has been reported using an offline HPTLC system [7]. This method has been used successfully to authenticate other species and commercial samples [8, 9, 10]. Herein, for the first time, the method has been adapted to a fully automated system that enables both the analysis of multiple samples in a sequence and the improvement of reproducibility using a “hands‐off” approach. Additionally, automated control of the gas phase further improved fingerprinting robustness. As shown in Figure 3, the identification of visible bands provides a qualitative assessment of the compounds in each sample. Under UV light (366 nm), the NP reagent highlighted the presence of phenolic compounds, particularly flavonoids, in the *Kalanchoe* species. Our findings show that quercetin‐type flavonoids (yellowish bands) were present in almost all fingerprints except in *K. daigremontiana* , which displays primarily kaempferol (greenish bands) in its fingerprint flavonoid profile. Subsequent derivatization with anisaldehyde reagent was performed in an attempt to identify additional features, but UV 366 nm prior to derivatization with NP reagent was determined to be the more comprehensive profiling method.

The comparison among the five *Kalanchoe* extract samples using HPTLC analysis revealed differences in their profiles, indicative of the presence of distinct markers across the species that were used for ID purposes (Figure 3). Chemical markers specific to each species are highlighted in bold. *K. daigremontiana* showed a fingerprint with a unique combination of four greenish zones (most likely kaempferol glycoside derivatives with different degrees of glycosylation based on comparison to standards) at ***R***_**F**_ **0.32**, 0.36 (faint), **0.41**, and 0.49 with extra faint zones above. Faint light bluish bands were observed from the application position up to *R*_F_ 0.2. *K*. x *houghtonii* displayed a fingerprint with faint light bluish bands from *R*_F_ 0.05 up to *R*_F_ 0.22 and then a broad bluish zone at ***R***_**F**_ **0.33**, followed by two faint yellowish bands at ***R***_**F**_ **0.37** and **0.42**, then at *R*_F_ **0.44** (bluish band), *R*_F_ 0.48 (greenish band), and at *R*_F_ 0.55 a couple of faint yellowish bands. *K. pinnata* showed bluish bands from *R*_F_ 0.05 up to *R*_F_ 0.22, two faint yellowish bands at ***R***_**F**_ **0.29** and **0.32**, followed by an intense yellowish band and ***R***_**F**_ **0.38**, then three faint bands at ***R***_**F**_ **0.42**, **0.43**, and 0.48, and a yellowish band at *R*_F_ 0.65. *K. marmorata* exhibited a fingerprint with bluish bands from the application position up to *R*_F_ 0.20. A broad intense yellowish zone was observed at ***R***_**F**_ **0.22**, then a bluish broad band at *R*_F_ 0.33, a medium intensity greenish band at *R*_F_ 0.36, followed by a couple of bands of medium intensity and intense at *R*_F_ 0.54 and ***R***_**F**_ **0.65**, respectively. *K. crenata* showed the most representative fingerprint with four yellowish bands at ***R***_**F**_ **0.16**, **0.32**, **0.44**, and **0.56**. Other important bands were observed at ***R***_**F**_ **0.48** (light bluish), ***R***_**F**_ **0.52** (faint greenish), ***R***_**F**_ **0.62** (greenish), and ***R***_**F**_ **0.71** (light bluish).

**FIGURE 4:**
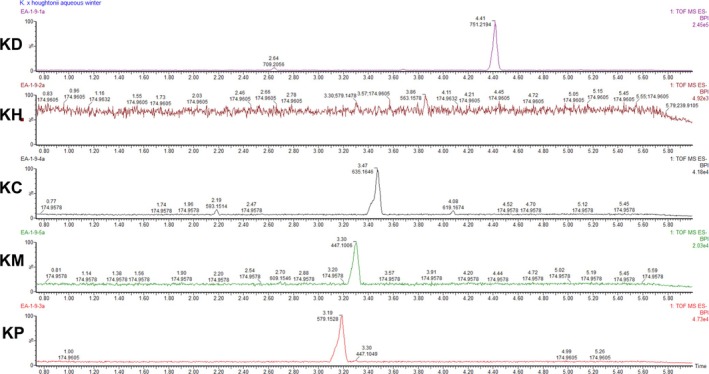
UPLC‐MS chromatograms of extracts of five *Kalanchoe* species in ESI negative ion mode (A). KD: *Kalanchoe daigremontiana* ; KH: *Kalanchoe* x *houghtonii*; KC: *Kalanchoe crenata* ; KM: *Kalanchoe marmorata* ; KP: *Kalanchoe pinnata* .

These findings align with previous research [37], which showed that *Kalanchoe* species are rich in bioactive phenolic compounds. A combinatorial approach using untargeted metabolomics and machine learning was applied by the authors to three *Kalanchoe* species ( *K. daigremontiana* , *Kalanchoe delagoensis* , and *K*. x *houghtonii*) to investigate the factors influencing the biosynthesis of these compounds. They concluded that these species contain distinct and diverse arrays of phenolic compounds, including flavonols, phenolic acids, and anthocyanins, as well as subfamilies such as catechols, lignans, stilbenes, flavones, flavanones, and flavanols. Evidence of these phenolic compounds has been suggested to further support therapeutic effects from the members of the *Kalanchoe* genus. To complement the HPTLC fingerprinting analysis, a comparative evaluation using reversed‐phase UPLC‐MS/MS^E^ in both positive (data not shown) and negative (Figure 4) ion modes was performed. Figure 4 displays the base peak intensity (BPI) chromatograms of *Kalanchoe* species extracts, and interestingly, extracts from each species were dominated by only one or two compounds that ionized above the baseline threshold. There were also no similarities between the major metabolites from each species. The same chromatographic peaks were observed in positive mode as corresponding [M + H]^+^ ions with no additional compounds above the baseline observed. Differences between the multiple band fingerprinting observed in the HPTLC profiling and the dominance of select compounds in UPLC‐MS analysis could be a result of several factors. As described above, HPTLC fingerprints shown in Figure 3 were recorded after a derivatization process with NP reagent, enhancing the detection of several minor compounds not observed before derivatization. These additional minor compounds may be present in the LCMS data but in abundances below the threshold for the included analyses. More detailed LC‐MS/MS metabolomics‐based analyses would likely reveal additional minor compounds and will be examined in future investigations. Additionally, the HPTLC fingerprinting was performed on normal phase plates and amenable solvents, whereas the UPLC‐MS profiling was performed with reversed‐phase chromatography and polar/aqueous solvents in which some of the more nonpolar compounds would not be amenable to chromatography and subsequent MS detection.

These findings are consistent with the research conducted previously [38], with chemical profiling performed using ultra‐high performance liquid chromatography (UHPLC) coupled with UV detection of extracts of *K. crenata* , *K. marmorata* , and *K. pinnata* , focusing on the presence of phenolic compounds, particularly flavonoids. The resulting chromatograms exhibited significant variations among the *Kalanchoe* species, as evidenced by their distinct compound profiles. A total of 14 major compounds (six from *K. daigremontiana* , four from *K. crenata* , three from *K. marmorata* , and one from *K. pinnata* ) were putatively identified and selected as possible chemical markers. The identification process involved analyzing the MS/MS fragmentation patterns combined with the available literature on the *Kalanchoe* genus (Table 3).

**TABLE 3:** Potential chemical markers for differentiation of *Kalanchoe* species.

Species	RT (min)	*m/z* [M − H]^−^	*m/z* [M + H]^+^	Tentative ID	References
KM	0.98	623.1360	625.1526	Quercetin glycoside derivative	[39]
KC	1.66	609.1445	611.1727	Quercetin 3‐galactoside 7‐rhamnoside	[39, 40]
KC	2.20	593.1514	595.1681	Kaempferol‐3‐O‐rutinoside or kaempferol 7‐O‐rutinoside	[39, 40]
KD	2.66	709.2056	711.2228	Sagittatin A	[39, 40, 41, 42, 43]
KM	2.70	609.1596	611.1626	Quercetin‐3‐O‐rutinoside (rutin)	[39]
KP	3.19	579.1528	581.1891	Quercetin glycoside derivative	[39]
KM	3.30	447.1006	449.1178	Quercetin 7‐O‐rhamnoside (vincetoxincoside b) or quercitrin	[40, 44]
KC	3.49	635.1646	637.1772	Kaempferol 3‐O‐(6″‐O‐acetyl)glucoside‐7‐O‐rhamnoside	[39, 40, 41, 42, 43]
KD	3.70	751.2138	753.2341	4″‐Acetylsagittatin A	[39, 40, 41, 42, 43]
KD	3.87	751.2194	753.2285	4″‐Acetylsagittatin A	[39, 40, 41, 42, 43]
KC	4.09	619.1673	621.1852	Sutchuenoside A	[39, 40]
KD	4.25	781.2288	783.2523	Kaempferol 3‐(2″‐rhamnosyl‐6″‐acetylgalactoside) 7‐rhamnoside	[39, 40, 41, 42, 43]
KD	4.44	751.2194	753.2341	4″‐Acetylsagittatin A	[39, 40, 41, 42, 43]
KD	5.06	823.2378	825.2578	Highly glycosylated flavonoids	[39, 41, 42, 43]

Abbreviations: KC: *Kalanchoe crenata* ; KD: *Kalanchoe daigremontiana* ; KM: *Kalanchoe marmorata* ; KP: *Kalanchoe pinnata* ; KH: *Kalanchoe* x *houghtoni*.

In the process of tentatively identifying the 14 compounds, it was important to align the given mass‐to‐charge (*m/z*) values for both [M − H]^−^ and [M + H]^+^ with published data of compounds typically identified in the *Kalanchoe* genus. Confidence in the putative identifications is enhanced based on their similarity to familiar phytochemicals found in *Kalanchoe* species and related bioactive phenolic compounds. MS^E^ fragmentation data revealed the presence of mass fragments with *m/z* [M − H]^−^: 301 and *m/z* [M + H]^+^: 303 for Compounds **1** ( *K. marmorata* , RT: 0.98 min), **2** ( *K. crenata* , RT: 1.66 min), **3** ( *K. crenata* , RT: 2.20 min), **5** ( *K. marmorata* , RT: 2.70 min), **6** ( *K. pinnata* , RT: 3.19 min), **7** ( *K. marmorata* , RT: 3.30 min), and **8** ( *K. crenata* , RT: 3.49 min), suggesting a quercetin core [45]. Additionally, mass fragments with *m/z* [M − H]^−^: 285 and *m/z* [M + H]^+^: 287 were observed for Compounds **4** ( *K. daigremontiana* , RT: 2.66 min), **9** ( *K. daigremontiana* , RT: 3.70 min), **10** ( *K. daigremontiana* , RT: 3.87 min), **11** ( *K. daigremontiana* , RT: 4.25 min), **12** ( *K. daigremontiana* , RT: 4.44 min), **13** ( *K. daigremontiana* , RT: 5.06 min), and **14**, suggesting a kaempferol core [46]. These retention times, along with the *m/z* values, strongly support the tentative identification of quercetin and kaempferol derivatives in the analyzed *Kalanchoe* species, consistent with compounds previously reported in the literature.

The compounds from the extracts of *K. marmorata* , *K. pinnata* , and those found in *K. crenata* with *m/z* [M − H]^−^: 609.1445, 593.1514, and 635.1646, [M + H]^+^: 611.1727, 595.1681, and 637.1772 may be representatives of quercetin glycoside derivatives, already described in a study with aqueous extracts of the species *Kalanchoe brasiliensis* and *K. pinnata* [39]. The compound with *m/z* [M − H]^−^: 593.1514, [M + H]^+^: 595.1681 present in the extract of *K. crenata* is suggestive of quercetin‐3‐O‐deoxyhexosyl(1‐2)deoxyhexoside, a compound previously described in flowers of the species *Kalanchoe blossfeldiana* [47].

The compound present in the extract of *K. marmorata* with *m/z* [M − H]^−^: 447.1006 and [M + H]^+^: 449.1178 potentially suggests a flavonol glycoside, possible quercetin 7‐O‐rhamnoside (vincetoxincoside b) or quercitrin [44]. The compound with *m/z* [M − H]^−^: 609.1596 and [M + H]^+^: 611.1626 present in the extract of *K. marmorata* is suggestive of a flavonol glycoside, possibly quercetin 3‐O‐rutinoside, known as rutin, based on the observed *m/z* values, which are consistent with literature reports [4, 38]. Additionally, the retention time of this compound indicates a more polar nature, further suggesting the presence of sugar moieties typically found in glycosides [48].

**FIGURE 5:**
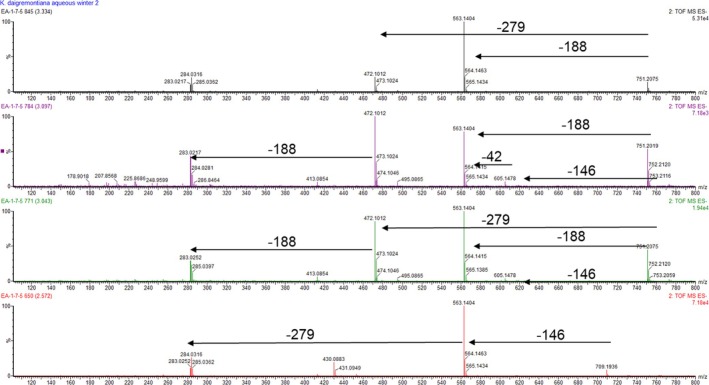
MS^E^ fragmentation of *Kalanchoe daigremontiana* compounds.

In all the compounds observed from the extract of *K. daigremontiana* and also for the compounds in *K. crenata* with *m/z* [M − H]^−^: 619.1673, [M + H]^+^: 621.1852, fragmentation data suggested the identification of kaempferol glycoside derivatives. Diagnostic fragmentation patterns observed for compounds in the extract of *K. daigremontiana* also imply glycosylated flavonoids or larger polyphenolic compounds [39, 41, 42, 43]. The MS^E^ fragmentation for compounds in *K. daigremontiana* 's extract is illustrated in Figure 5.

It is important to note that there are no reports in the literature identifying the observed masses for *K. daigremontiana*. However, these masses likely correspond to the major compounds of the species. Therefore, we interpreted the observed mass fragments by referencing available literature on other *Kalanchoe* species and other plants that contain phenolic compounds. The loss of [M‐42]^−^ indicates the removal of an acetate group. The loss of [M‐146]^−^ reflects the loss of a deoxy‐hexose unit. The loss of [M‐162]^−^ indicates the removal of a hexose unit. The loss of [M‐188]^−^ is associated with an acetyl‐deoxy‐hexose group. Finally, the loss of [M‐279]^−^ suggests the fragmentation of a diglycosyl unit. These losses are consistent with structures of flavonoids and glycosylated compounds previously described in the literature [42, 43, 47].

Flavonoids including quercetin and kaempferol derivatives have been identified and reported in aqueous leaf extracts of *K. daigremontiana* and *K. pinnata* [35, 49]. These results contribute to the understanding of phenolic compounds in *Kalanchoe* species, supporting the significant medicinal potential of this genus. Further identification efforts will require confirmation through comparison with standards and detailed spectroscopic analysis of purified compounds.

### 3.3 Quality Control of Commercial Samples

In the pharmaceutical and herbal medicine industry, microscopic and phytochemical analyses are essential to ensure the quality and safety of final products. The importance of such evaluations is particularly underscored in the analysis of *Kalanchoe* extracts and purportedly related commercial products where several discrepancies are apparent across multiple analytical techniques.

**FIGURE 6:**
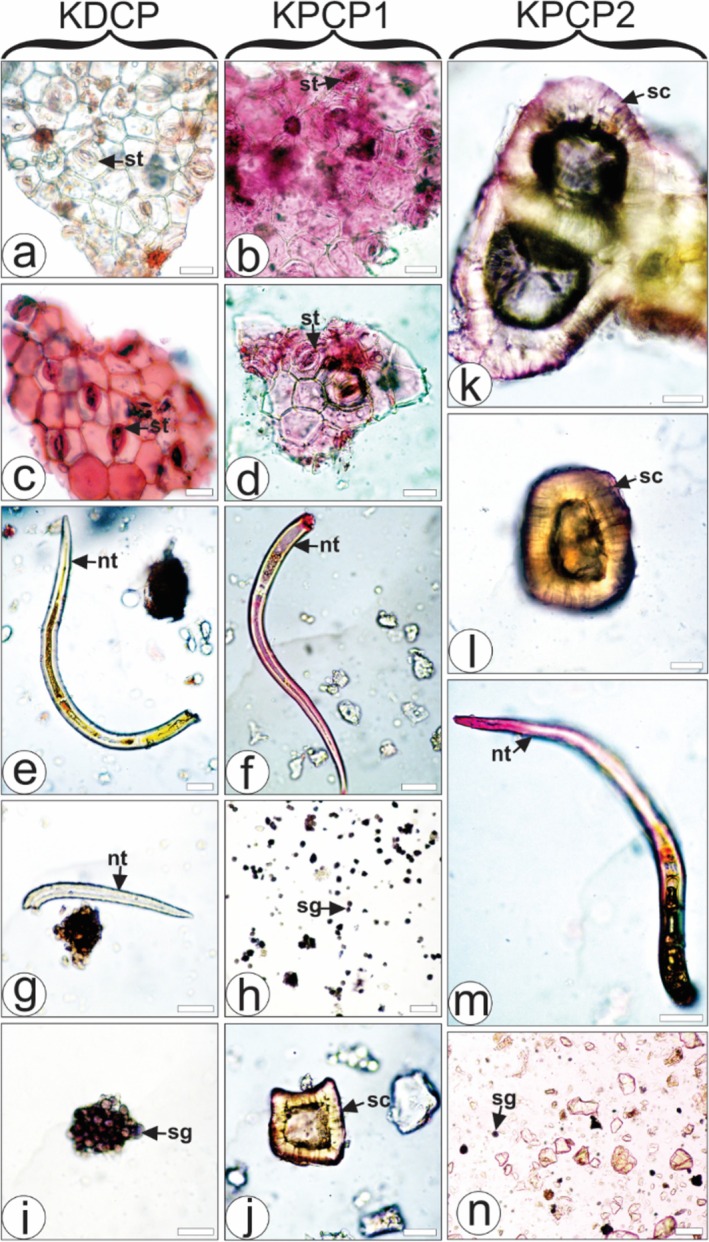
Microscopical analysis of commercially available products from *Kalanchoe* species. *Kalanchoe daigremontiana* commercial product (KDCP), *Kalanchoe pinnata* commercial product 1 (KPCP1), and *Kalanchoe pinnata* commercial product 2 (KPCP2). Astra blue and basic fuchsine (b,d,e–g,j,k–m); safranin (a,c); iodine solution (h,i,n). nt: nonglandular trichome; sc: sclereid cell; sg: starch grain; st: stomata. Scale bar: 25 μm (b–g,i–m); 50 μm (a,n); 100 μm (h).

During the pharmacobotanical visual analysis of authentic material versus commercial products (Figure 6), numerous inconsistencies were noted. In the KDCP, the characteristics of thick and straight anticlinal epidermal cell walls, as well as paracytic stomata (Figure 6a,c), and simple nonglandular trichome (Figure 6e,g) were observed. These features differ from those found in the authenticated material (Figure 2b,g and Table 2). Although starch grains are also detected in the KDCP sample (Figure 6i), they were previously detected in *K. daigremontiana* [49].

In the KPCPA, thick and straight anticlinal epidermal cell walls (Figure 6b,d), simple nonglandular trichome (Figure 6f), and sclereid cells (brachysclereids) were observed (Figure 6j). However, these characteristics were not found in the authenticated material (Figure 2d,i and Table 2). Furthermore, polyhedral starch grains were found in the KPCPA sample (Figure 6h). These starch grains are similar to those commonly found in *Zea mays* L. (Poaceae) [50], suggesting potential contamination or substitution with maize/corn starch, which is unexpected in *K. pinnata* products. This raises concerns about the authenticity of the product and highlights issues that warrant further investigation.

In the second KPCPB, simple nonglandular trichome (Figure 3m), sclereid cells (brachysclereids) (Figure 6k,l), and rounded starch grains (Figure 6n) were observed. Although nonglandular trichomes and brachysclereids were present, they were not found in the authenticated material (Figure 2d,i,n,v–x,e′ and Table 2).

**FIGURE 7:**
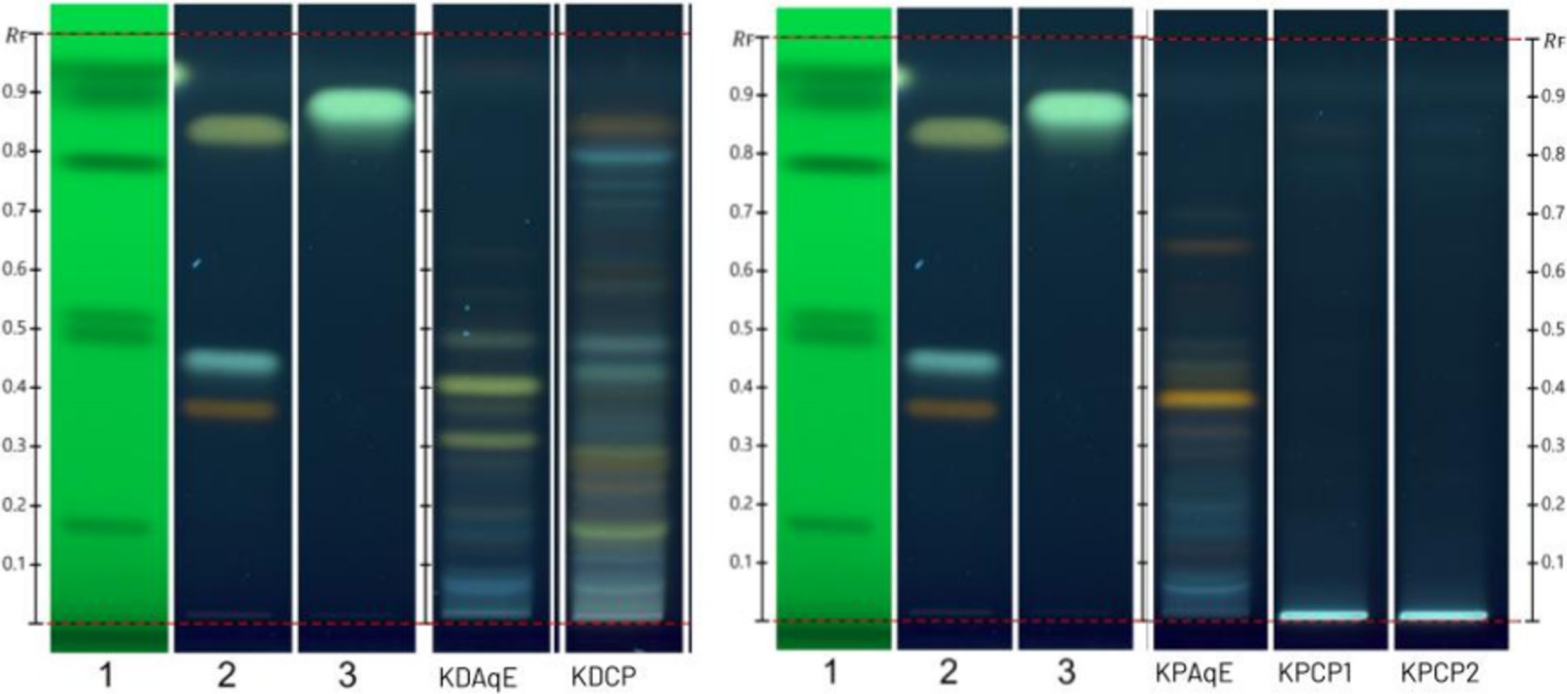
HPTLC profile of *Kalanchoe* species extracts and commercial products under 366 UV light after derivatization with NP reagent. Track 1: SST; Track 2: rutin, chlorogenic acid, and quercetin (with increasing *R*_F_); Track 3: kaempferol; KDAqE: aqueous extract of *K. daigremontiana* ; KPAqE: aqueous extract of *K. pinnata* ; KDCP: commercial product of *K. daigremontiana* ; KPCP1: Brand 1 commercial product of *K. pinnata* ; KPCP2: Brand 2 commercial product of *K. pinnata* . Contrast 2.0.

To complement the microscopic analyses and further assess the quality and authenticity of a feedstock, combining microscopic observations with phytochemical analysis is essential. To assess verified *Kalanchoe* species and corresponding commercial products, a chromatographic evaluation was developed. HPTLC fingerprinting (Figure 7) further highlighted discrepancies between the commercial products and authenticated material, providing insights into authenticity and quality. Analysis of commercial products stated to be prepared from *K. daigremontiana* and *K. pinnata* had markedly different metabolite profiles from the respective extracts. Consequently, the authenticity and proper sourcing of the *Kalanchoe* species in commercial samples could not be validated because they did not match the respective plant extracts.

**FIGURE 8:**
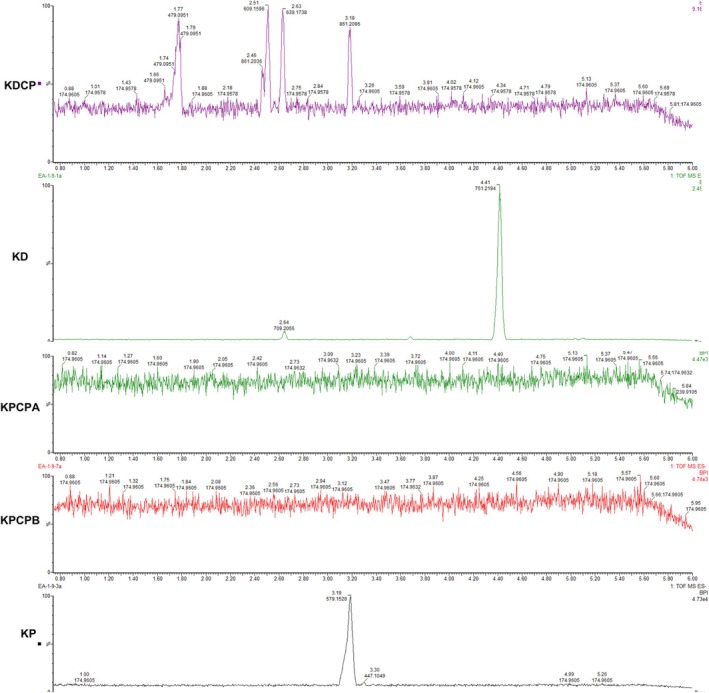
Chromatograms of aqueous extracts of *Kalanchoe daigremontiana* and *Kalanchoe pinnata* and their related commercial products. KD: *Kalanchoe daigremontiana* ; KP: *Kalanchoe pinnata* ; KDCP: *Kalanchoe daigremontiana* ; KPCPA: *Kalanchoe pinnata* commercial product brand A; KPCPB: *Kalanchoe pinnata* commercial product brand B.

Supportive examination of the UPLC‐MS chromatograms from the commercial products showed a complete lack of major metabolites present in the *Kalanchoe* extract samples, even when the commercial products were tested at a 10× concentration compared with the authenticated extracts (Figure 8).

There are a variety of possible explanations for the discrepancies observed when comparing the commercial products to authentic standards including differences in plant handling (drying of plant material prior to extraction has been shown to greatly reduce phenolic content) [51]. However, based on these analyses, adulterants were detected in all three commercial samples (KDCP, KPCP1, and KPCP2) tested. Worldwide, the amount of adulterated herbal products varies significantly across continents [52]. This variation reflects the complexity of assessing herbal product authenticity [53], as different regions employ varying methodologies and have different levels of regulation. The high prevalence of adulteration calls for orthogonal approaches [54], incorporating both chemical and microscopic methods, to ensure the quality and safety of herbal products in global markets.

As has been seen with many other botanicals, the findings described here for *Kalanchoe* extracts and related commercial products confirm the need for comprehensive characterization and quality control in the production of herbal medicines derived from *Kalanchoe* species. Differences in the composition of NPs can be attributed to several factors, including agricultural practices, methods of extraction, preparation techniques, and storage conditions, which can contribute to variations in the compound content between different batches, potentially influencing the physiological or pharmacological activity of the products. Valuable tools such as HPTLC and untargeted metabolomic analysis can improve the quality of these products [10, 55].

This study highlights the effectiveness of combining pharmacobotanical analysis, UPLC‐MS/MS^E^ metabolome profiling, and HPTLC fingerprinting in identifying and differentiating *Kalanchoe* species, their extracts, and commercial products. This integrated approach is crucial for ensuring the quality, safety, and consistency of herbal products. It marks the first comparison of metabolome differences among *Kalanchoe* species, emphasizing the importance of such analytical methods in quality control and standardization within the herbal product industry to meet higher product quality and consumer satisfaction.

## Data Availability

The data that support the findings of this study are available from the corresponding author upon reasonable request.

## References

[1] Brazil. National Health Surveillance Agency (ANVISA) , “Collegiate Board Resolution ‐ RDC No. 26, of May 13, 2014. Provides for the registration of herbal medicines and the registration and notification of traditional herbal products,” Off Gaz Union 1 (2014): 35. Accessed August 20, 2024, https://bvsms.saude.gov.br/bvs/saudelegis/anvisa/2014/rdc0026_13_05_2014.html.

[2] United States Congress . “Dietary Supplement Health and Education Act of 1994,” Public Law No. 103–417, 108 Stat. 4325.

[3] J. Manfron , “Farmacobotânica: uma ferramenta importante para a detecção de adulterações em matérias‐primas vegetais,” in *A Farmacognosia no Brasil* [e‐book]: memórias da sociedade brasileira de farmacognosia, 1st ed., ed. L. C. Baratto (Petrópolis, RJ: Ed. do Autor, 2021): 191–202.

[4] E. Assis de Andrade , I. Machinski , A. C. Terso Ventura , et al., “A Review of the Popular Uses, Anatomical, Chemical, and Biological Aspects of Kalanchoe (Crassulaceae): A Genus of Plants Known as “Miracle Leaf”,” Molecules 28, no. 14 (2023): 5574. 37513446 10.3390/molecules28145574 PMC10383218

[5] USP . “General Chapter <1064> Identification of Articles of Botanical Origin by High‐Performance Thin‐layer Chromatography,” In: USP 38 – NF 33 (2015).

[6] CAMAG . “Application fields,” Retrieved August 20, 2024, from https://www.camag.com/applications/application‐fields.

[7] W. H. Perera , D. A. Frommenwiler , M. H. M. Sharaf , and E. Reich , “An Improved High‐Performance Thin‐Layer Chromatographic Method to Unambiguously Assess *Ginkgo biloba* Leaf Finished Products,” Journal of Planar Chromatography‐‐Modern TLC 34, no. 6 (2021): 559–560.

[8] K. A. Antunes , L. M. Monteiro , C. Howard , et al., “Comprehensive High‐Performance Thin‐Layer Chromatography Analysis of *Monteverdia ilicifolia* Leaf and Its Adulterants,” Natural Product Research (2023), 10.1080/14786419.2023.2289080. 38073526

[9] A. Parveen , J. S. Adams , V. Raman , et al., “Comparative morpho‐Anatomical and HPTLC Profiling of *Tinospora* Species and Dietary Supplements,” Planta Medica 86, no. 7 (2020): 470–481. 32168549 10.1055/a-1120-3711

[10] V. Raman , Y. H. Wang , S. G. Saroja , et al., “Characterization of *Calea ternifolia* and Its Adulterant *Chromolaena odorata* Using Macro‐Microscopy, HPTLC and UHPLC‐UV–MS,” Revista Brasileira de Farmacognosia 33 (2023): 790–801.

[11] J. Abidi , S. Ammar , S. Ben Brahim , K. Skalicka‐Woźniak , Z. Ghrabi‐Gammar , and M. Bouaziz , “Use of Ultra‐High‐Performance Liquid Chromatography Coupled With Quadrupole‐Time‐Of‐Flight Mass Spectrometry System as Valuable Tool for an Untargeted Metabolomic Profiling of *Rumex tunetanus* Flowers and Stems and Contribution to the Antioxidant Activity,” Journal of Pharmaceutical and Biomedical Analysis 162 (2019): 66–81. 30223144 10.1016/j.jpba.2018.09.001

[12] M. M. Hurkul , A. Cetinkaya , S. Yayla , and S. A. Ozkan , “Advanced Sample Preparation and Chromatographic Techniques for Analyzing Plant‐Based Bioactive Chemicals in Nutraceuticals,” Journal of Chromatography Open 5 (2024): 100131.

[13] L. Perez de Souza , S. Alseekh , F. Scossa , and A. R. Fernie , “Ultra‐High‐Performance Liquid Chromatography High‐Resolution Mass Spectrometry Variants for Metabolomics Research,” Nature Methods 18 (2021): 733–746. 33972782 10.1038/s41592-021-01116-4

[14] R. S. Plumb , K. A. Johnson , P. Rainville , et al., “UPLC/MS(E); a new Approach for Generating Molecular Fragment Information for Biomarker Structure Elucidation,” Rapid Communications in Mass Spectrometry 20, no. 13 (2006): 1989–1994. 16755610 10.1002/rcm.2550

[15] D. A. Johansen , Plant Microtechnique (New York: McGraw‐Hill, 1940), 523 p.

[16] G. P. Berlyn and J. P. Miksche , Botanical Microtechnique and Cytochemistry (Ames, IA: Iowa State University Press, 1976).

[17] K. R. Roeser , “Die Nadel der Schwarzkiefer‐Massenprodukt und Kunstwerk der Natur,” Mikrokosmos 61 (1972): 33–36.

[18] T. P. O'Brien , N. Feder , and M. E. McCully , “Polychromatic Staining of Plant Cell Walls by Toluidine Blue O,” Protoplasma 59 (1964): 368–373.

[19] C. Fuchs , “Fuchsin Staining With NaOH Clearing for Lignified Elements of Whole Plants or Plant Organs,” Stain Technology 38, no. 3 (1963): 141–144.

[20] T. K. T. Do , M. Schmid , M. Phanse , et al., “Development of the First Universal Mixture for use in System Suitability Tests for High‐Performance Thin Layer Chromatography,” Journal of Chromatography. A 1638 (2021): 461830. 33453655 10.1016/j.chroma.2020.461830

[21] USP , “High‐Performance Thin Layer Chromatography procedure for identification of articles of botanical origin,” In: United States Pharmacopeia and National Formulary USP40–NF35. United States Pharmacopoeial Convention, (2017) Chapter 203 258–260.

[22] Z. Akulova‐Barlow , “Kalanchoe,” Cactus and Succulent Journal 81 (2009): 268–276.

[23] S. Herrando‐Moraira , D. Vitales , N. Nualart , et al., “Global Distribution Patterns and Niche Modeling of the Invasive *Kalanchoe* × *houghtonii* (Crassulaceae),” Scientific Reports 10 (2020): 3143. 32081991 10.1038/s41598-020-60079-2 PMC7035272

[24] C. Liu , C. Zhu , and H. M. Zeng , “Key KdSOC1 Gene Expression Profiles During Plantlet Morphogenesis Under Hormone, Photoperiod, and Drought Treatments,” Genetics and Molecular Research 15, no. 1 (2016): 1–14. 10.4238/gmr.15017579 26909971

[25] M. Chernetskyy , A. Woźniak , A. Skalska‐Kamińska , et al., “Structure of Leaves and Phenolic Acids in *Kalanchoe daigremontiana* Raym.‐Hamet & H. Perrier,” Acta Sci pol Hortorum Cultus. 17 (2018): 137–155.

[26] R. Zawirska‐Wojtasiak , B. Jankowska , P. Piechowska , and S. M. Szkudlarz , “Vitamin C and Aroma Composition of Fresh Leaves From Kalanchoe Pinnata and Kalanchoe Daigremontiana,” Scientific Reports 9 (2019): 19786. 31875020 10.1038/s41598-019-56359-1 PMC6930271

[27] A. Q. Majaz , M. Khurshid , and S. Nazim , “The Miracle Plant (*Kalanchoe pinnata*): A Phytochemical and Pharmacological Review,” Int J Res Ayurveda Pharm. 2 (2011): 1478–1482.

[28] M. Bhatti , A. Kamboj , A. K. Saluja , and U. K. Jain , “In Vitro Evaluation and Comparison of Antioxidant Activities of Various Extracts of Leaves and Stems of Kalanchoe Pinnatum,” International Journal of Green Pharmacy 6 (2012): 340–347.

[29] S. Bhavsar , B. Dhru , M. Zaveri , and D. Chandel , “A Comparative Pharmacognostical and Phytochemical Analysis of *Kalanchoe pinnata* (Lam.) Pers. Leaf Extracts,” Journal of Pharmacognosy and Phytochemistry 7 (2018): 1519–1527.

[30] M. Chernetskyy and E. Weryszko‐Chmielewska , “Structure of *Kalanchoë pumila* Bak. Leaves (Crassulaceae DC.),” Acta Agrobotanica 61 (2008): 29–37.

[31] N. S. Moreira , L. B. S. Nascimento , M. V. Leal‐Costa , and E. S. Tavares , “Comparative Anatomy of Leaves of *Kalanchoe pinnata* and *K. crenata* in sun and Shade Conditions, as a Support for Their Identification,” Revista Brasileira de Farmacognosia 22 (2012): 929–936.

[32] H. S. Abdel‐Raouf , “Anatomical Traits of Some Species of *Kalanchoe* (Crassulaceae) and Their Taxonomic Value,” Annals of Agricultural Science 57, no. 1 (2012): 73–79.

[33] M. R. Duarte and C. C. Zaneti , “Morfologia de folhas de bálsamo: *Sedum dendroideum* Moc. et Sessé ex DC, Crassulaceae,” Rev Lecta. 20 (2002): 153–160.

[34] C. R. Metcalfe and L. Chalk , Anatomy of the Dicotyledons, vol. 1 (Oxford: Clarendon Press, 1950): 243–245.

[35] P. García‐Pérez , E. Lozano‐Milo , M. Landin , and P. P. Gallego , “From Ethnomedicine to Plant Biotechnology and Machine Learning: The Valorization of the Medicinal Plant *Bryophyllum* sp,” Pharmaceuticals 13 (2020): 444. 33291844 10.3390/ph13120444 PMC7762000

[36] L. B. S. Nascimento , L. M. Casanova , and S. S. Costa , “Bioactive Compounds From *Kalanchoe* Genus Potentially Useful for the Development of new Drugs,” Life 13 (2023): 646. 36983802 10.3390/life13030646 PMC10058616

[37] P. García‐Pérez , L. Zhang , B. Miras‐Moreno , et al., “The Combination of Untargeted Metabolomics and Machine Learning Predicts the Biosynthesis of Phenolic Compounds in *Bryophyllum* Medicinal Plants (Genus *Kalanchoe*),” Plants. 10 (2021): 2430. 34834793 10.3390/plants10112430 PMC8620224

[38] I. Machinski , E. A. Andrade , V. M. Schaffka , et al., “Exploring the Pharmacognostical and Phytochemical Profiles of Aqueous Extracts of *Kalanchoe* ,” Chemistry & Biodiversity 21, no. 7 (2024): e202400660. 38771297 10.1002/cbdv.202400660

[39] E. R. D. de Araujo , J. Félix‐Silva , J. B. Xavier‐Santos , et al., “Local Anti‐Inflammatory Activity: Topical Formulation Containing *Kalanchoe brasiliensis* and *Kalanchoe pinnata* Leaf Aqueous Extract,” Biomedicine & Pharmacotherapy 113 (2019): 108721. 30856538 10.1016/j.biopha.2019.108721

[40] L. Souza , J. P. S. O. Oliveira , A. S. Fernandes , A. F. Macedo , C. F. Araujo‐Lima , and I. Felzenszwalb , “UHPLC‐MS Metabolomic Profile and in Silico Pharmacokinetic Approach of *Kalanchoe daigremontiana* Raym.‐Hamet & H. Perrier Aqueous Extracts,” Journal of Pharmaceutical and Biomedical Analysis 238 (2024): 115827. 37951139 10.1016/j.jpba.2023.115827

[41] J. Stefanowicz‐Hajduk , M. Asztemborska , M. Krauze‐Baranowska , et al., “Identification of Flavonoids and Bufadienolides and Cytotoxic Effects of *Kalanchoe daigremontiana* Extracts on Human Cancer Cell Lines,” Planta Medica 86 (2020): 239–246. 31994149 10.1055/a-1099-9786

[42] F. G. Ürményi , G. D. Saraiva , L. M. Casanova , et al., “Anti‐HSV‐1 and HSV‐2 Flavonoids and a new Kaempferol Triglycoside From the Medicinal Plant Kalanchoe Daigremontiana,” Chemistry & Biodiversity 13 (2016): 1707–1714. 27472283 10.1002/cbdv.201600127

[43] M. Beszterda and R. Frański , “Electrospray Ionization Mass Spectrometric Behavior of Flavonoid 5‐O‐Glucosides and Their Positional Isomers Detected in the Extracts From the Bark of *Prunus cerasus* L. and *Prunus avium* L.,” Phytochemical Analysis 32, no. 3 (2021): 433–439. 32929795 10.1002/pca.2991

[44] Y. Xiao , W. Zheng , X. Lu , Y. Wu , H. Chen , and X. Zhang , “Identification of Multiple Constituents in Shuganjieyu Capsule and rat Plasma After Oral Administration by Ultra‐Performance Liquid Chromatography Coupled With Electrospray Ionization Quadrupole Time‐Of‐Flight Tandem Mass Spectrometry,” Molecules 21 (2016): 1275.27669205

[45] G. Chen , H. Hu , L. Wang , X. Li , S. Huang , and L. Zhang , “Analysis of Flavonoids in *Rhamnus davurica* and Its Antiproliferative Activities,” Molecules 21 (2016): 1275. 27669205 10.3390/molecules21101275 PMC6273673

[46] D. Tsimogiannis , V. Oreopoulou , N. Kalogeropoulos , et al., “Characterization of Flavonoid Subgroups and Hydroxy Substitution by HPLC‐MS/MS,” Molecules 12 (2007): 593–606. 17851414 10.3390/12030593 PMC6149353

[47] A. H. Nielsen , C. E. Olsen , and B. L. Møller , “Flavonoids in Flowers of 16 *Kalanchoë blossfeldiana* Varieties,” Phytochemistry 66 (2005): 2829–2835. 16297414 10.1016/j.phytochem.2005.09.041

[48] S. Kumar and A. K. Pandey , “Chemistry and Biological Activities of Flavonoids: An Overview,” Scientific World Journal 2013 (2013): 162750. 24470791 10.1155/2013/162750 PMC3891543

[49] E. A. Andrade , I. Machinski , V. P. Almeida , et al., “Differentiating two Species of ‘Mother‐Of‐Thousands’: *Kalanchoe Daigremontiana* and *Kalanchoe* x *houghtonii* ,” Brazilian Archives of Biology and Technology 67 (2024): e24240333.

[50] SBFGnosia , “Brazilian Society of Pharmacognosy,” Amidos. Retrieved 17 April 2024, http://www.sbfgnosia.org.br/Ensino/amido.html.

[51] A. Snoussi , I. Essaidi , H. Ben Haj Koubaier , et al., “Drying Methodology Effect on the Phenolic Content, Antioxidant Activity of *Myrtus communis* L. Leaves Ethanol Extracts and Soybean oil Oxidative Stability,” BMC Chemistry 15 (2021): 31. 33952328 10.1186/s13065-021-00753-2 PMC8097818

[52] M. C. Ichim , “The DNA‐Based Authentication of Commercial Herbal Products Reveals Their Globally Widespread Adulteration,” Frontiers in Pharmacology 10 (2019): 1227. 31708772 10.3389/fphar.2019.01227 PMC6822544

[53] M. C. Ichim and A. Booker , “Chemical Authentication of Botanical Ingredients: A Review of Commercial Herbal Products,” Frontiers in Pharmacology 12 (2021): 666850. 33935790 10.3389/fphar.2021.666850 PMC8082499

[54] M. C. Ichim , A. Häser , and P. Nick , “Microscopic Authentication of Commercial Herbal Products in the Globalized Market: Potential and Limitations,” Frontiers in Pharmacology 11 (2020): 876. 32581819 10.3389/fphar.2020.00876 PMC7295937

[55] L. Mattoli , M. Gianni , and M. Burico , “Mass Spectrometry‐Based Metabolomic Analysis as a Tool for Quality Control of Natural Complex Products,” Mass Spectrometry Reviews 42 (2023): 1358–1396. 35238411 10.1002/mas.21773

